# Ozone promotes macrophage efferocytosis and alleviates neuropathic pain by activating the AMPK/Gas6-MerTK/SOCS3 signaling pathway

**DOI:** 10.3389/fimmu.2024.1455771

**Published:** 2024-11-19

**Authors:** Shirong Ruan, Rumeng Jia, Liang Hu, Yuge Liu, Qingyan Tian, Kunmao Jiang, Xinyue Xia, Xueyou Tao, Wen-Tao Liu, Yinbing Pan, Fan Hu

**Affiliations:** ^1^ Department of Pharmacology, School of Basic Medical Sciences, Nanjing Medical University, Nanjing, Jiangsu, China; ^2^ Department of Anesthesiology, Ili and Jiangsu Joint Institute of Health, The Friendship Hospital of Ili Kazakh Autonomous Prefecture, Yining, Xinjiang, China; ^3^ Department of Anesthesiology, Yangzhou Maternal and Child Health Hospital Affiliated to Medical College of Yangzhou University, Yangzhou, Jiangsu, China; ^4^ Department of Anesthesiology, The First Affiliated Hospital of Nanjing Medical University, Nanjing, China; ^5^ State Key Laboratory of Reproductive Medicine and Offspring Health, Nanjing Medical University, Nanjing, Jiangsu, China

**Keywords:** neuropathic pain, ozone, macrophage, efferocytosis, MerTK, SOCS3, neuroinflammation

## Abstract

**Background:**

Neuropathic pain (NPP) is a multifaceted pain syndrome that occurs as a consequence of physical injury or underlying diseases, with an incidence rate of 7%-10%, NPP poses a significant clinical challenge as current treatment options are ineffective. The accumulation of apoptotic cells and neuroinflammation play crucial roles in the pathological mechanisms of NPP. Here, we aim to investigate strategies for effectively clearing apoptotic cells and provide therapeutic interventions for NPP.

**Methods:**

CCI mice were treated with different concentrations of ozone (15μg, 30μg, 45μg) to investigate the effects on the accumulation of apoptotic cells and neuroinflammation. In vitro, the phagocytic function of BMDM towards apoptotic neutrophils after ozone treatment was examined.

**Results:**

We found ozone at a concentration of 30μg significantly alleviated mechanical hypersensitivity in CCI mice and ozone significantly upregulates the phagocytic activity of BMDM. Furthermore, we investigated the mechanisms and found ozone could activate AMPK, upregulate Gas6 (but not Protein S), activate MerTK (a key receptor involved in apoptosis), and enhance the phagocytic function of BMDM towards apoptotic neutrophils. It caused the promotion of SOCS3 expression and the suppression of inflammatory factors IL-1β, IL-6, and TNF-a. Interestingly, the effect of ozone in alleviating CCI-induced pain was abolished by the AMPK inhibitor CC and the MerTK receptor inhibitor UNC2541.

**Conclusion:**

Ozone facilitated macrophage clearance of apoptotic cells, decreased neuroinflammation by activation of p-AMPK/Gas6/MerTK/SOCS3 signaling pathway, which may become an effective therapeutic approach for neuropathic pain after further clinical validation.

## Introduction

Neuropathic pain is characterized by spontaneous pain, abnormal pain sensations, sensory abnormalities, and hyperalgesia with lack of effective treatment options (1). Recommended medications for neuropathic pain include tricyclic antidepressants, serotonin-norepinephrine reuptake inhibitors, pregabalin, gabapentin, etc., as the first-line drugs. Second-line drugs include tramadol, capsaicin, etc. Strong opioids and type A botulinum toxin are considered third-line options (2). However, the first-line medications are associated with numerous side effects, while the use of second-line opioid drugs is limited due to their tolerance and addictive properties, significantly impacting patients’ quality of life (3, 4). Therefore, it is imperative to explore the pathogenic mechanisms and therapeutic targets for neuropathic pain.

Apoptosis is a form of cell death in which the cell actively participates in its demise. It plays a crucial role in various physiological activities and is also involved in diseases such as cancer, other chronic conditions, autoimmune disorders, and neurodegenerative diseases (5, 6). Studies have shown that peripheral nerve injury leads to significant cell death, and during this process, dying cells release DAMPs. Additionally, bacterial infection during tissue damage produces PAMPs, triggering neuroinflammatory responses and upregulating the expression of inflammatory factors such as IL-1β, TNF-α, and INF-γ (7–10). The activation of resident immune cells further results in substantial infiltration of immune cells, collectively releasing inflammatory mediators including IL-1β, TNF-α, kinins, substance P, calcitonin gene-related peptide (CGRP), nerve growth factor (NGF), and prostaglandin E (PGE), forming an “inflammatory soup” (11). On the other hand, the inflammatory infiltration releases a large number of pro-inflammatory cytokines, further promoting apoptotic responses in cells, exacerbating neuropathic pain reactions (12). Therefore, during nerve injury, the accumulation of apoptotic cells and neuroinflammation induction may be critical pathological mechanisms of neuropathic pain.

Efferocytosis, also known as cell burial, is the process by which phagocytes engulf apoptotic cells, thereby clearing them in a programmed manner within cellular biology. The clearance of apoptotic cells (ACs) requires phagocytes to express receptors capable of recognizing ligands associated with ACs. This enables phagocytes to undergo cytoskeletal rearrangement upon binding to ACs, leading to the formation of phagosomes and lysosome fusion for the degradation of ACs. Additionally, efferocytosis not only clears apoptotic cells but also exerts anti-inflammatory and tissue repair effects by reducing the expression of inflammatory factors (13). When efferocytosis is impaired, these functions are disrupted, resulting in aggravated inflammation, impaired resolution, and disease development. The TAM receptors (TYRO3, AXL, MerTK) serve as classical efferocytic receptors and require bridging molecules such as Gas6 and protein S to recognize phosphatidylserine (PS) signals released by apoptotic cells, thereby initiating efferocytosis (14). Studies have indicated that MerTK, a receptor tyrosine kinase, plays a crucial role in the engulfment and effective clearance of apoptotic cells (15, 16). However, the underlying mechanisms remain unclear.

Ozone is an allotrope of oxygen, characterized by its fishy odor and pale blue color. It possesses strong oxidative properties and can undergo oxidation reactions at relatively low temperatures. The use of “ozone therapy” can be traced back to the World War I when German soldiers used ozone to treat gas gangrene. Ozone’s predominant effects include spectrum sterilization, anti-inflammatory action, and promotion of local tissue regeneration. Due to its small molecular size, ozone has excellent penetration ability, allowing it to rapidly infiltrate cells and tissues to eliminate pathogenic microorganisms. Simultaneously, it generates oxygen, which can be absorbed and utilized by cells and tissues to facilitate repair. Currently, ozone therapy is used in the clinical treatment of various diseases, including osteoarthritis, soft tissue pain, and peripheral arterial circulation disorders (17). Ozone autohemotherapy, by leveraging the effects of ozone, improves the body’s oxygen metabolism and eliminates excessive free radicals to achieve therapeutic outcomes. Ozone therapy has also demonstrated positive effects in the treatment of COVID-19 patients (18). Studies have shown that intrathecal injection of ozone can alleviate neuropathic pain in rats through the GluR6-NF-κB/p65 signaling pathway (19). Our previous research has also revealed ozone could treat neuropathic pain by activating AMPK (20). However, the relationship between ozone and the efferocytic function of macrophages remains unclear and requires further investigation.

Here, we demonstrated that ozone, as an emerging therapeutic approach, could effectively treat NPP by promoting macrophage efferocytosis, reducing the accumulation of apoptotic cells and suppressing neuroinflammatory responses. These findings proposed new strategies for alleviating NPP and provide strong evidence for the clinical translation of ozone therapy.

## Methods

### Animals

Adult male SPF-grade ICR mice and C57BL/6 mice, weighing 20-22g and aged 6-8 weeks, were obtained from the Experimental Animal Center of Nanjing Medical University. The license number for the use of experimental animals is SYXK (Su) 2021-0023.

### Reagents and antibodies

The following reagents and antibodies were used in the study: β-actin antibody from ABclonal (China), p-AMPK antibody from Cell Signaling Technology (USA), SOCS3 antibody and Protein S antibody from Santa Cruz Biotechnology (USA), Gas6 antibody from Abcam (USA), p-MerTK antibody from Thermo Fisher Scientific(USA), AICAR from Selleckchem (USA), Compound C from MedChemExpress (USA), UNC2541 from Topscience (China), BCECF from APExBIO (USA), Secondary antibodies for Western blot from Sigma (USA). Secondary antibodies for immunofluorescence from Jackson Immunoresearch Laboratories (USA). HiScript^®^ RT SuperMix and SYBR Green were purchased from Vazyme (China). Fetal bovine serum (FBS) from Biological Industries (BI, USA). Other cell culture media and supplements from Kaiki Creature (China).

### Chronic constriction injury model

Mice were anesthetized with sodium pentobarbital and sciatic nerve was ligatured by 4-0 chromic gut sutures. After 14 days, the CCI model was established. Mice were anesthetized with pentobarbital sodium, and the sciatic nerve was exposed; however, the nerve was not ligated with 4-0 chromic gut sutures to serve as the control group.

### Preparation of medical ozone

The oxygen tank was connected to the Herrmann medical ozone generator to prepare medical ozone (a mixture of ozone and oxygen) with different masses according to experimental requirements (15μg, 30μg, 45μg). Following the successful establishment of the CCI model, mice were administered intraperitoneal injections of varying concentrations of ozone once daily for a continuous duration of 7 days.

### Doses of agonists/inhibitors used in experiments

#### Cellular level

BMDM cells were extracted from WT mice and treated with LPS (1 µg/ml) for 6 hours to establish an inflammation model. The cells were pretreated with AICAR (300 µM), CC (20 µM) for 15 minutes and UNC2541 (2.5 µM) for 30 minutes. After treatment with medical ozone (30 µg/ml) for 4 hours, subsequent experiments were conducted to evaluate the relevant indicators.

#### Animal level

After establishing the mouse CCI model for 14 days, mice received CC (100μg) or UNC2541 (25μM) pretreatment around the sciatic nerve half an hour in advance. Subsequently, ozone (30μg, i.p.) was administered for 7 days.

### Mouse behavioral testing

Mechanical sensitivity was detected using Von Frey Hairs (Woodland Hills, Los Angeles, CA, USA). The 0.16, 0.4, 0.6, 1.0, 1.4, 2.0 of the von Frey hairs were used. Animals were placed in boxes set on an elevated metal mesh floor and allowed to habituate for 30 minutes before testing. The plantar surface of each hind paw was stimulated with a series of von Frey hairs with logarithmically increasing stiffness perpendicular to the plantar surface. Each mouse was tested three times, and the average threshold was measured.

### Western blot

Mouse plasma and sciatic nerve samples were collected and prepared for SDS-PAGE electrophoresis. After transfer, the membrane was blocked with blocking solution (5% BSA + 5% milk in TBST) at room temperature for 2 hours. Primary antibodies were incubated (p-AMPK, 1:1000; Gas6, 1:1000; proteinS, 1:5000; p-ADAM17, 1:1000; SOCS3, 1:1000; SOCS1, 1:1000; p-MerTK, 1:1000) at 4°C for 16 hours. Subsequently, secondary antibodies (1:5000) were incubated at room temperature for 2 hours. A Molecular Imager (ChemiDoc, Bio-Rad) was used for image capture. BMDM cell total protein and supernatant were collected and prepared as samples. Western blot experiments were conducted following the steps above.

### Extraction of bone marrow-derived macrophages

Mouse tibia and femur were aseptically isolated. Cells were flushed into RPMI 1640 medium, filtered through a 70μm nylon mesh to remove debris, and centrifuged at 1500 rpm for 5 minutes. The cell pellet was collected and resuspended in DMEM medium (10% FBS + 10% L929 culture medium + 1% penicillin-streptomycin solution, 100×), and then cultured. After 7 days, subsequent experiments were conducted. Macrophage polarization into M1 type was induced by stimulating the cells with 100 ng/ml LPS and 50 ng/ml IFN-γ for 24 hours.

### Extraction of bone marrow neutrophils

Mouse tibia and femur were aseptically isolated. Cells were washed with 0.9% saline. The cell suspension was centrifuged at 1200g for 5 minutes at 4°C and cell pellet was resuspended in 3 ml of saline. Then, 9 ml of histopaque-1077 was added into a 50 ml centrifuge tube, and the 3 ml cell suspension was carefully layered on top. The tube was centrifuged at 2000g for 20 minutes at 4°C without brakes. Cell pellet was resuspended in 5 ml of saline, which was layered on 10 ml of histopaque-1119. The tube was centrifuged at 2000g for 20 minutes at 4°C without brakes. The intermediate layer, which contained neutrophils, was collected. Neutrophils were cultured in a medium (10% serum + 1% antibiotics in RPMI 1640 medium) for subsequent experiments.

### Induction of apoptosis in bone marrow neutrophils

After extracting bone marrow neutrophils according to the above procedure, they were resuspended in PBS. The neutrophils were then exposed to ultraviolet (UV) irradiation for 15 minutes to induce apoptosis. Apoptotic neutrophils were labeled with BCECF (1mg/ml) for 30 minutes. The cells were centrifuged at 1500rpm for 5 minutes, and the cell pellet was collected and resuspended in PBS. The cells were used for subsequent experiments.

### Immunofluorescence

Fourteen days after establishing the CCI mouse model, mice were anesthetized with sodium pentobarbital (40mg/kg.i.p.). The mice were perfused with 4% paraformaldehyde. The L4-L5 spinal cord segments and the dorsal root ganglia (DRG) were collected frozen sectioned. After washing with PBS three times, blocking solution (0.3% Triton-100 + 10% donkey serum) was added and incubated at room temperature for 2 hours. Primary antibodies (dilution ratios and sources are as follows: c-GRP, 1:200, rabbit; cleaved-caspase3, 1:300; F4/80, 1:50, mouse) were incubated at 4°C for 16 hours. Fluorescent secondary antibodies (1:300) were incubated at room temperature for 2 hours. The sections were observed under an LSM800 confocal microscope for image acquisition and analysis.

### Cellular immunofluorescence

BMDMs were extracted according to the above method. An inflammation model was established by stimulating BMDMs with LPS, and medical ozone was used for treatment. BMDMs were treated with CC (20mM, pre-treated for 15 minutes), UNC2541 (2.5mM, pre-treated for 30 minutes), and AICAR (300mM, pre-treated for 15 minutes) respectively. After adding the treated apoptotic neutrophils, co-cultivation was carried out for 15 minutes. The samples were observed under an LSM800 confocal microscope for image acquisition and analysis.

### Sciatic nerve electron microscopy imaging

After establishing the CCI mouse model for 14 days, the mice were anesthetized with pentobarbital sodium (40mg/kg, i.p.) and perfused with 4% paraformaldehyde. The sciatic nerve segment was collected and pre-fixed in glutaraldehyde for preservation at 4°C. Subsequently, the samples were washed three times for 30 minutes each with phosphate-buffered saline, transferred to osmium tetroxide for two hours for post-fixation, and then washed three times for 10 minutes each with buffer solution. The samples underwent dehydration with ethanol and acetone. The samples were then immersed in embedding resin. Subsequent processing involved sectioning and slide preparation, followed by image acquisition and analysis using transmission electron microscopy.

### RT-PCR

The sciatic nerve of mice and BMDM cells were collected. Total RNA was extracted using TRIzol reagent. The mRNA was reverse transcribed into cDNA using xHiscript II qRT supermix in a 10 μL reaction system, and SYBR Green and primers were added for fluorescence quantitative analysis using an RT-PCR instrument. The relative expression levels of Il1b, Il6, and Tnfa were calculated and quantified using the 2-ΔΔCt method after normalization with a reference gene (beta-actin). All the primers used in this study are listed in **Supplementary Data Sheet 1** (**Supplementary Table S1**).

### Data processing and analysis

GraphPad Prism 8.0 software (GraphPad Software, San Diego, CA, USA) was used to conduct all the statistical analyses. All data are presented as mean ± S.E.M. Differences between groups were analyzed using analysis of variance (ANOVA), including one-way and two-way ANOVA, combined with t-test (Student-Newman-Keuls Test). A p-value of less than 0.05 was considered statistically significant, while a p-value of less than 0.01 indicated a highly significant difference.

## Results

### The excessive accumulation of apoptotic cells and neuroinflammation are important pathological mechanisms of neuropathic pain

**Figure 1:**
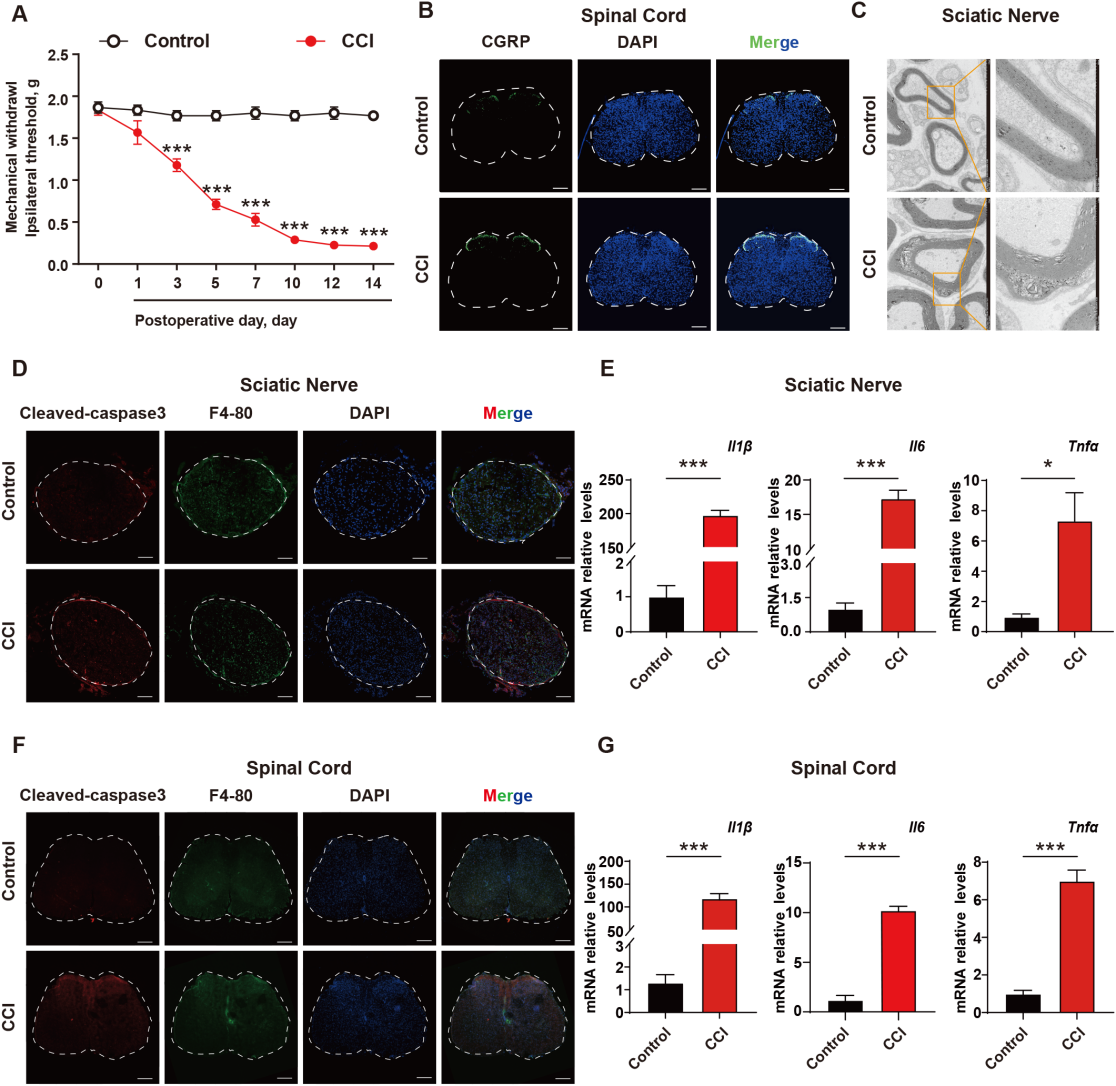
The excessive accumulation of apoptotic cells and neuroinflammation are important pathological mechanism of neuropathic pain. **(A)** CCI mouse model was established and the mechanical pain threshold of mice was measured using Von Frey Test (n=6). **(B)** On the 14th day after establishing the CCI mouse model, immunofluorescence was used to examine the content of c-GRP in the dorsal horn of the spinal cord (n=3). Scale bars, 200μm. **(C)** Mouse sciatic nerves were collected to investigate the demyelination response in CCI mice (n=3). **(D)** Apoptotic cell accumulation in mouse sciatic nerves was detected using immunofluorescence (n=3). Scale bars, 100μm. **(E)** q-PCR was used to detect the transcription levels of inflammatory factors in mouse sciatic nerves (n=3). **(F, G)** Mouse spinal cord was collected to examine the accumulation of apoptotic cells and transcription levels of inflammatory factors (n=3). Scale bars, 200μm. Significant differences were revealed following unpaired Student’s t-test **(E, G)** or Two-way ANOVA **(A)** (*p < 0.05, **p < 0.01, and ***p < 0.001 *vs*. Control).

To investigate the role of apoptotic cells and neuroinflammation in neuropathic pain, a CCI mouse model was established. The Von Frey test was used to evaluate mechanical pain thresholds in mice, and the results showed that CCI mice exhibited mechanical hypersensitivity compared to control group (**Figure 1A**). Spinal cords were collected from the mice, and it was found that the c-GRP content in the dorsal horn of the spinal cord was increased in CCI mice (**Figure 1B**). Transmission electron microscopy was used to observe the sciatic nerve in mice, revealing significant demyelination reactions in the sciatic nerve of CCI mice (**Figure 1C**). To further explore the mechanism, the accumulation of apoptotic cells in the sciatic nerve and spinal cord were investigated using cleaved-caspase 3 (as a marker for apoptotic cells), as well as levels of inflammatory factors. The results showed that CCI could induce a significant increase in apoptotic cells, with apparent infiltration of macrophages around them and elevated transcription levels of the inflammatory factors IL-1β, TNF-α, and INF-γ (**Figures 1D–G**, **Supplementary Figure S1**) These results suggested that in the CCI mice, there was an excessive accumulation of apoptotic cells and induction of neuroinflammation, which were important pathological mechanisms in NPP.

### Ozone decreases the accumulation of apoptotic cells and suppresses neuroinflammation to treat NPP

**Figure 2:**
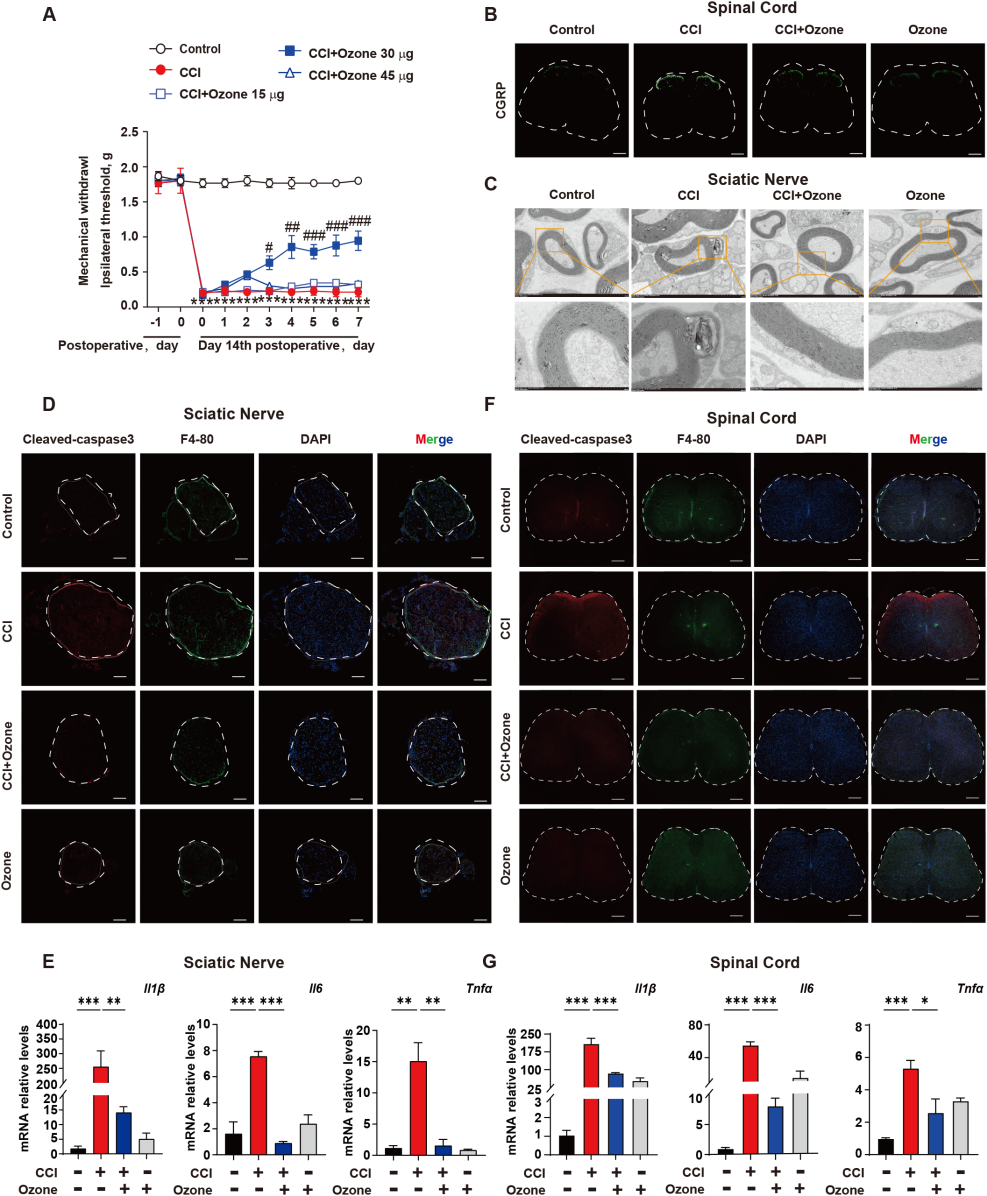
Ozone decreases the accumulation of apoptotic cells and suppresses neuroinflammatory responses to treat NPP. **(A)** On the 14th day after establishing the CCI mouse model, mice were exposed to ozone at different concentrations for 7 days, and the effect of ozone on the mechanical pain threshold of CCI mice was evaluated (n=6). **(B)** On the 7th day of ozone treatment, the content of c-GRP in the dorsal horn of the spinal cord was detected (n=3), Scale bars, 200μm; mouse sciatic nerves were collected to investigate the effect of ozone on the demyelination response in CCI mice (n=3) **(C)**. **(D)** Apoptotic cell accumulation in the sciatic nerves among different treatment groups was examined (n=3). Scale bars, 100μm. **(E)** Transcription levels of inflammatory factors in mouse sciatic nerves were assessed (n=3). **(F, G)** The effect of ozone on apoptotic cells in the spinal cord of CCI mice and the impact on transcription levels of inflammatory factors were examined (n=3). Scale bars, 200μm. Significant differences were revealed following one-way ANOVA **(E, G)** or two-way ANOVA **(A)** (*p < 0.05, **p < 0.01, and ***p < 0.001; ^#^p < 0.05, ^##^p < 0.01, and ^###^p < 0.001 *vs*. CCI).

Ozone therapy has been widely used in the treatment of various conditions including COVID-19, skin diseases, and pain management (21–23). Our previous studies have shown promising therapeutic effects of ozone in chemotherapy-induced enteritis and neuropathic pain (20, 24). To further investigate the mechanism of ozone in NPP, we administered continuous intraperitoneal injections of ozone at concentrations of 15 μg, 30 μg, and 45 μg to mice. The results revealed that ozone at a concentration of 30 μg significantly alleviated mechanical hypersensitivity in CCI mice (**Figure 2A**). Notably, administering 30 µg of ozone alone did not affect the mechanical pain threshold in mice (**Supplementary Figure S5**). In addition to conducting the von Frey test, we further evaluated the heat pain threshold in mice using the hot plate test. The results demonstrated that CCI mice exhibited significant heat hyperalgesia, while ozone treatment exerted a notable alleviating effect (**Supplementary Figure S4**). Interestingly, ozone at a concentration of 30 μg significantly reduced the accumulation of c-GRP in the dorsal horn of the spinal cord in CCI mice (**Figure 2B**), and inhibited demyelination reactions in the sciatic nerve (**Figure 2C**). Further investigations showed that ozone could reduce the accumulation of apoptotic cells in the sciatic nerve and the spinal cord and suppressed the inflammatory response induced by apoptotic cells (**Figures 2D–G**, **Supplementary Figure S2**). These results suggested ozone could decrease the accumulation of apoptotic cells and suppress neuroinflammatory responses for the treatment of NPP.

### Ozone promotes macrophage efferocytosis of apoptotic cells through the activation of AMPK/gas6/MerTK pathway

**Figure 3:**
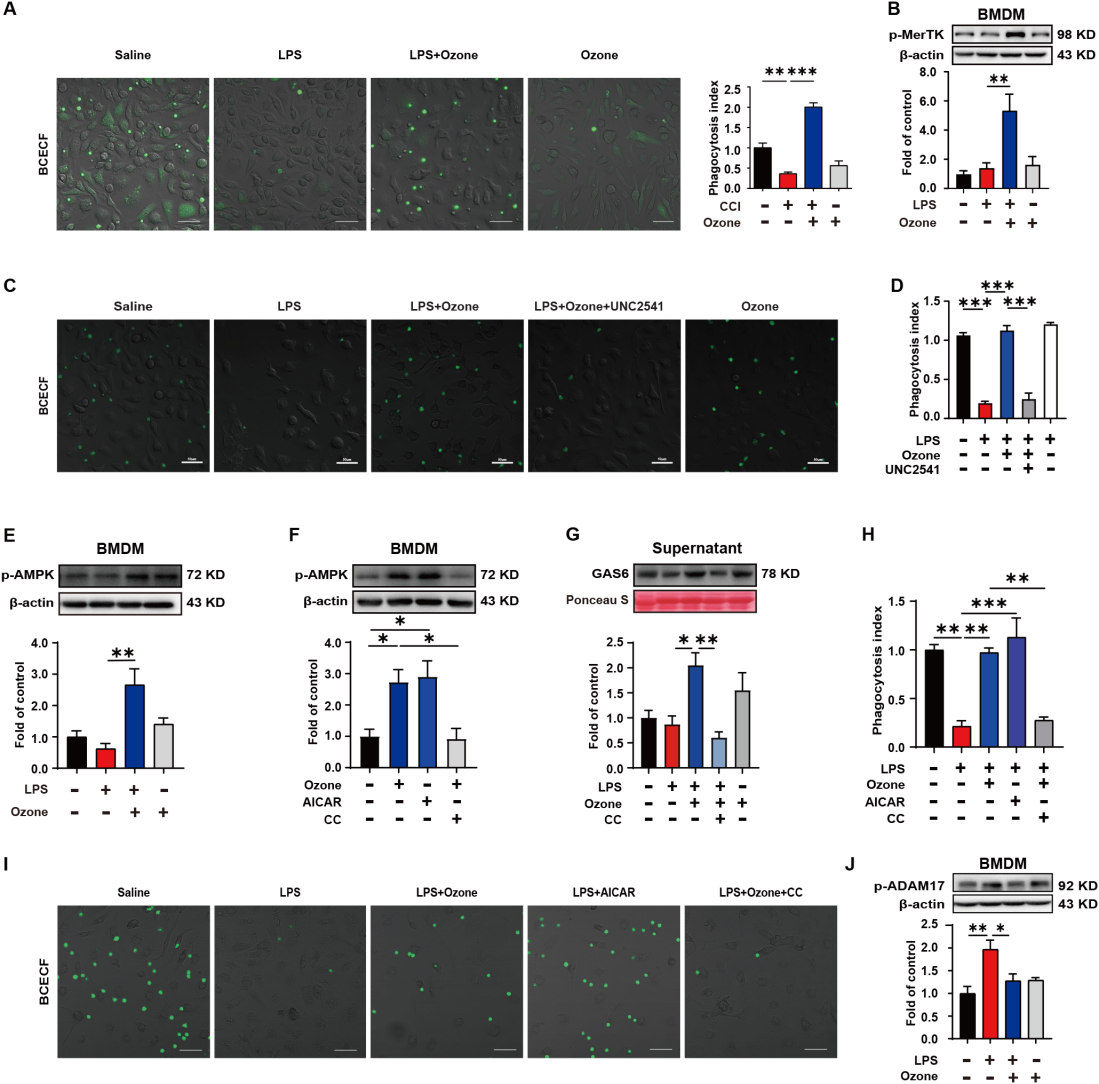
Ozone promotes macrophage efferocytosis of apoptotic cells through the activation of AMPK/gas6/MerTK pathway. **(A)** BMDMs from WT mice were stimulated with LPS (1μg/ml) for 6 hours to establish an inflammatory model and exposed to ozone (30μg/ml) for 4 hours. The engulfment of apoptotic neutrophils by BMDMs was evaluated (n=3). Scale bars, 50μm. **(B)** Whole-cell lysates of BMDMs were collected to examine the expression level of p-MerTK (n=3). **(C, D)** BMDMs were stimulated with LPS (1μg/ml) for 6 hours to establish an inflammatory model and pretreated with a MerTK receptor inhibitor UNC2541 (2.5mM) for 30 minutes, followed by treatment with ozone (30μg/ml) for 4 hours. The engulfment of apoptotic neutrophils by BMDMs was evaluated (n=3). Scale bars, 50μm. **(E, F)** Whole-cell lysates of BMDMs were collected to detect the expression level of p-AMPK and BMDMs were stimulated with an AMPK inhibitor CC (20μM) for 15 minutes to investigate its effect on ozone-induced AMPK activation (n=3). **(G)** Supernatants of BMDMs were collected to measure the secretion of Gas6 in different treatment groups (n=3). **(H, I)** BMDMs were stimulated with LPS (1μg/ml) for 6 hours to establish an inflammatory model and pretreated with AICAR (300μM) or CC (20μM) for 15 minutes, followed by treatment with ozone (30μg/ml) for 4 hours. The engulfment of apoptotic neutrophils by BMDMs was evaluated (n=3). Scale bars, 50μm. **(J)** The expression level of p-ADAM17 of BMDM was detected (n=3). Significant differences were revealed following one-way ANOVA **(B, D, E-H, J)** (*p < 0.05, **p < 0.01, and ***p < 0.001).

In order to explore the mechanism by which ozone suppresses the accumulation of apoptotic cells, the BMDM were extracted from WT mice and stimulated with LPS (1 μg/ml) for 6 hours to establish an inflammatory model, simulating the neuroinflammatory response *in vivo*. Apoptotic neutrophils were used to mimic the excessive accumulation of apoptotic cells. The phagocytic clearance ability of BMDMs towards apoptotic neutrophils was assessed, and the results showed that LPS-stimulated BMDMs exhibited significantly dysfunction of phagocytosis towards apoptotic neutrophils, while ozone could promote the efferocytosis of apoptotic cells by BMDMs (**Figure 3A**). We also examined the phagocytic activity of normal RAW264.7 cells toward apoptotic neutrophils and found that RAW264.7 cells do not phagocytose these apoptotic neutrophils (**Supplementary Figure S3**). Therefore, we will focus on the BMDMs of macrophages. We further explored the mechanism by which ozone promotes macrophage efferocytosis. Our results showed that the expression of phosphorylated Mer receptor tyrosine kinase (p-MerTK) was significantly increased in BMDM treated by ozone compared to LPS stimulation, indicating that ozone could activate the efferocytic receptor, MerTK (**Figure 3B**). To further investigate the crucial role of MerTK in NPP, BMDMs were pre-treated with the MerTK inhibitor UNC2541. Remarkably, UNC2541 abolished pro-phagocytic effect of ozone and significantly inhibited the engulfment of apoptotic neutrophils by BMDMs (**Figures 3C, D**). We further found that LPS stimulation inhibited AMPK activation, while ozone treatment significantly upregulated the expression of p-AMPK (**Figure 3E**). Stimulation of BMDMs with the AMPK activator AICAR also upregulated p-AMPK, and this effect was reversed when the AMPK inhibitor CC was used, canceling the ozone-induced activation of AMPK (**Figure 3F**). Additionally, Growth arrest-specific 6(Gas6), an important bridging molecule for MerTK receptor, has been reported to be associated with AMPK activation (25, 26). Measurement of Gas6 levels in the cell culture supernatant revealed that ozone upregulated the expression of Gas6, which was also canceled by CC treatment (**Figure 3G**). The relationship between AMPK activation and MerTK-mediated macrophage efferocytosis was performed. The results showed that LPS stimulation led to impaired efferocytic function in BMDMs, while ozone treatment reversed this condition. AICAR simulated the effect of ozone by promoting the engulfment and clearance of apoptotic neutrophils by BMDMs. However, the AMPK inhibitor CC canceled the ozone-induced enhancement of efferocytosis (**Figures 3H, I**). An additional movie file shows this in more detail (see **Supplementary Data Sheet 1**, **Supplementary Videos S1–S8**). On the other hand, proteolytic shedding of MerTK receptor by proteases such as a disintegrin and metalloproteinase domain-containing protein 17 (ADAM17) could lead to dysfunction of efferocytosis in inflammatory macrophages and the generation of soluble Mer fragments. These soluble fragments bound with the bridging molecule GAS6, preventing the interaction between GAS6 and intact MerTK receptors (27–30). ADAM17 is the main protease responsible for cleaving MerTK, which may be the reason for the decreased expression of p-MerTK in BMDM stimulated by LPS. We examined the expression levels of phosphorylated ADAM17 (p-ADAM17) and found ozone significantly reduced the levels of p-ADAM17 (**Figure 3J**), suggesting that ozone may also upregulate MerTK by inhibiting ADAM17-mediated cleavage of MerTK. Overall, these findings suggested that ozone promoted macrophage efferocytosis of apoptotic cells mediated by MerTK by activating the AMPK/Gas6 pathway and inhibiting ADAM17 activation, treating neuropathic pain.

### Ozone suppresses neuroinflammation by upregulating SOCS3 expression

**Figure 4:**
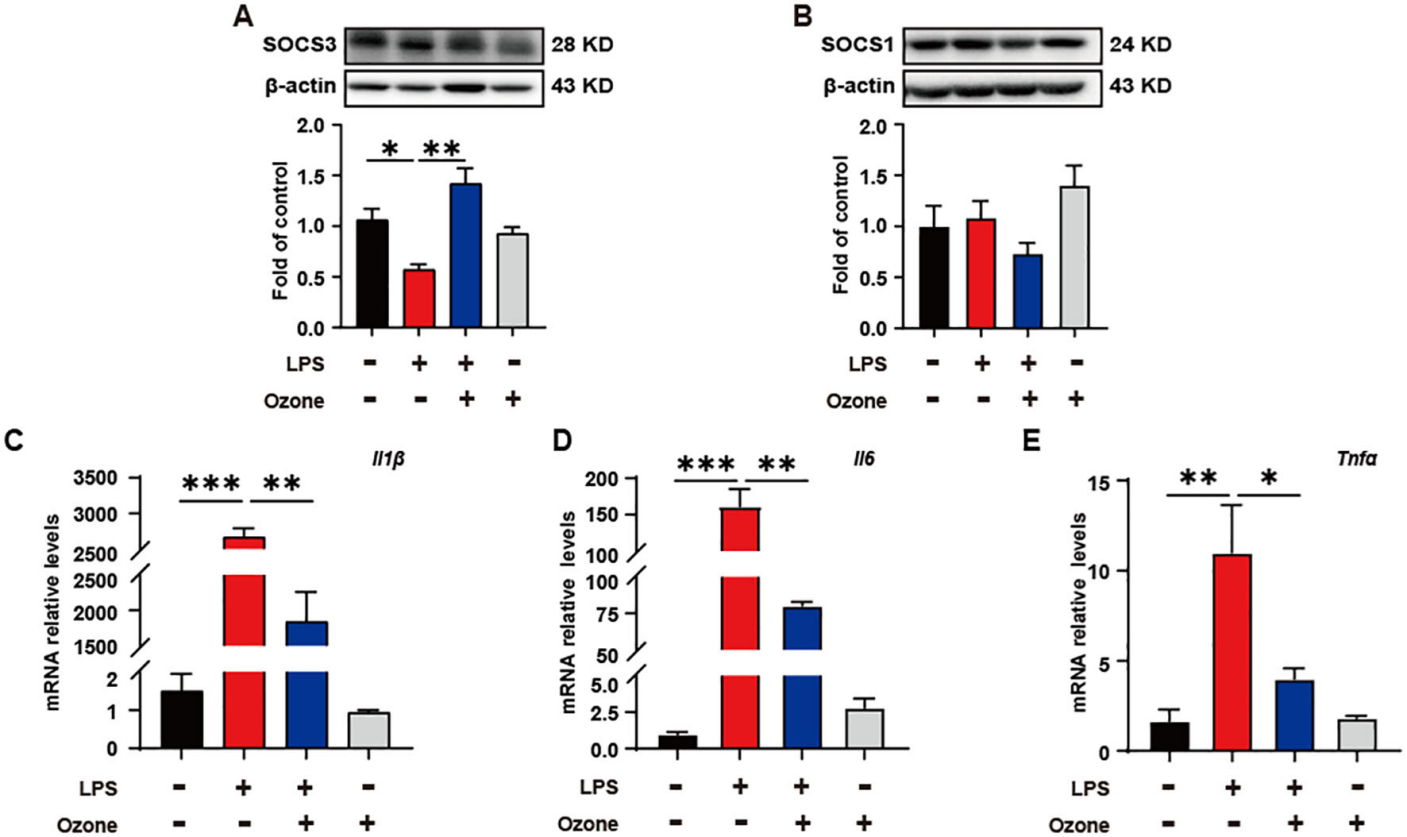
Ozone suppresses neuroinflammatory responses by upregulating SOCS3 expression. **(A, B)** The SOCS3 and SOCS1 expression in BMDM were examined (n=3). **(C-E)** qPCR was performed to detect the transcription levels of inflammatory cytokines in BMDMs (n=3). Significant differences were revealed following one-way ANOVA **(A–E)** (*p < 0.05, **p < 0.01, and ***p < 0.001).

In order to verify the impact of ozone on inflammatory responses during the process of efferocytosis, the expression levels of two inflammation brake factors, SOCS3 and SOCS1, were examined. The results showed that ozone promoted the expression of SOCS3, rather than SOCS1 (**Figures 4A, B**). Suppressor of cytokine signaling (SOCS) proteins are a family of proteins that respond to various cytokines and growth factor signals by inducing the attenuation of cytokine transduction through multiple mechanisms, thus negatively regulating innate and adaptive immune responses (31). Studies have shown that efferocytic receptors upregulate the ubiquitin ligase inhibitors of cytokine signaling 1 (SOCS1) and SOCS3, thereby blocking IFNα-mediated signal transducers and activators of transcription 1 (STAT1) signaling and the expression of pro-inflammatory genes (32, 33). To investigate the effect of SOCS3 in inflammation, the transcript levels of inflammatory cytokines in BMDMs were examined. As expected, LPS led to a significant increase in the mRNA levels of inflammatory cytokines IL-1β, IL-6, and TNF-a, while ozone pretreatment significantly reduced the release of IL-1β, IL-6, and TNF-a (**Figure 4C**). These results suggest that ozone promotes the anti-inflammatory effects primarily through upregulating SOCS3.

### Ozone alleviates neuropathic pain by activating the AMPK/Gas6-MerTK/SOCS3 signaling pathway

**Figure 5:**
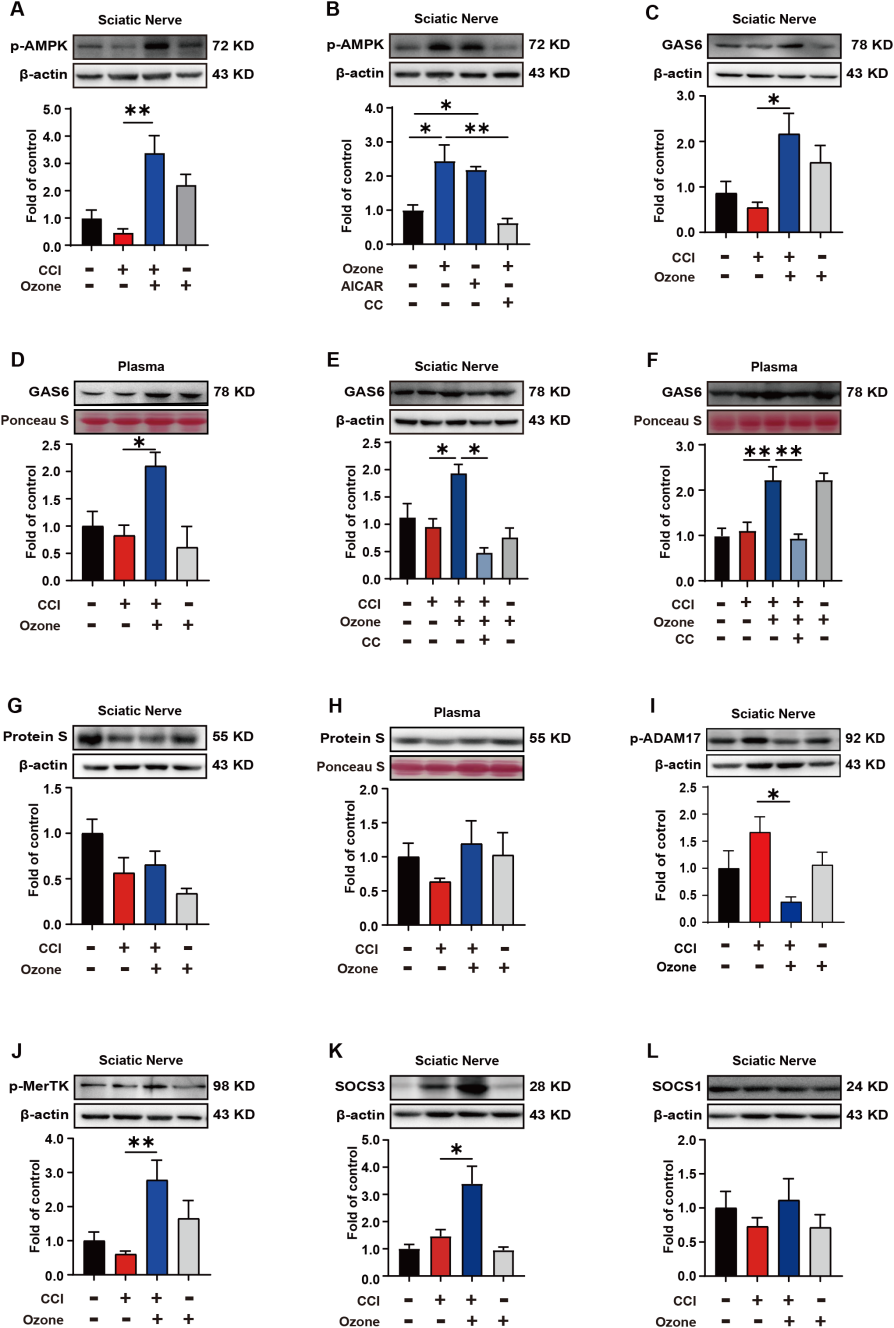
Ozone alleviates neuropathic pain by activating the AMPK/Gas6-MerTK/SOCS3 signaling pathway. **(A)** After establishing the mouse CCI model for 14 days, ozone (30μg, i.p.) was administered for 7 days. The sciatic nerves of mice were collected to measure the p-AMPK expression level. **(B)** Mice received AICAR (0.25mg/kg) and CC (100μg) pretreatment around the sciatic nerve half an hour in advance. Ozone (30μg, i.p.) was administered for 7 days, and the sciatic nerves were collected to investigate p-AMPK levels (n=6). **(C, D)** Western blot was used to examine the Gas6 expression of sciatic nerves and plasma in CCI mice (n=6). **(E, F)** After establishing the mouse CCI model for 14 days, mice received CC (100μg) pretreatment around the sciatic nerve half an hour in advance. Ozone (30μg, i.p.) was administered for 7 days, and the expression levels of Gas6 in the sciatic nerves and plasma were detected (n=6). **(G, H)** Western blot was used to measure the expression levels of Protein S (n=6). **(I, J)** p-ADAM17 and p-MerTK expression in the sciatic nerves were detected by western blot (n=6). **(K, L)** The expression levels of SOCS3 and SOCS1 in the sciatic nerves were examined (n=6). Significant differences were determined by one-way ANOVA **(A-L)**. (*p < 0.05, **p < 0.01, and ***p < 0.001).

*In vivo*, we further validated the mechanism by which ozone alleviates neuropathic pain. The results showed that ozone significantly activated AMPK and upregulated the expression level of p-AMPK in the sciatic nerve compared to the CCI group, while CC abolished this effect of ozone (**Figures 5A, B**). Further analysis of Gas6 levels in the sciatic nerve and plasma revealed that ozone markedly promoted Gas6 expression (**Figures 5C, D**). Interestingly, CC abolished this effect of ozone once again (**Figures 5E, F**). Besides Gas6, Protein S is also an essential bridging molecule for MerTK receptor. We investigated the Protein S expression in the sciatic nerve and plasma and unfortunately found that ozone did not significantly active on Protein S (**Figures 5G, H**). This suggested ozone mainly upregulated MerTK through Gas6 rather than Protein S. Additionally, we examined the levels of p-ADAM17 in the sciatic nerve, and the results were consistent with the experiments *in vitro*. Ozone inhibited ADAM17 expression (**Figure 5I**), ultimately upregulated p-MerTK (**Figure 5J**). Finally, we also found that ozone could promote the expression of SOCS3 in the sciatic nerve rather than SOCS1 (**Figures 5K, L**).

**Figure 6:**
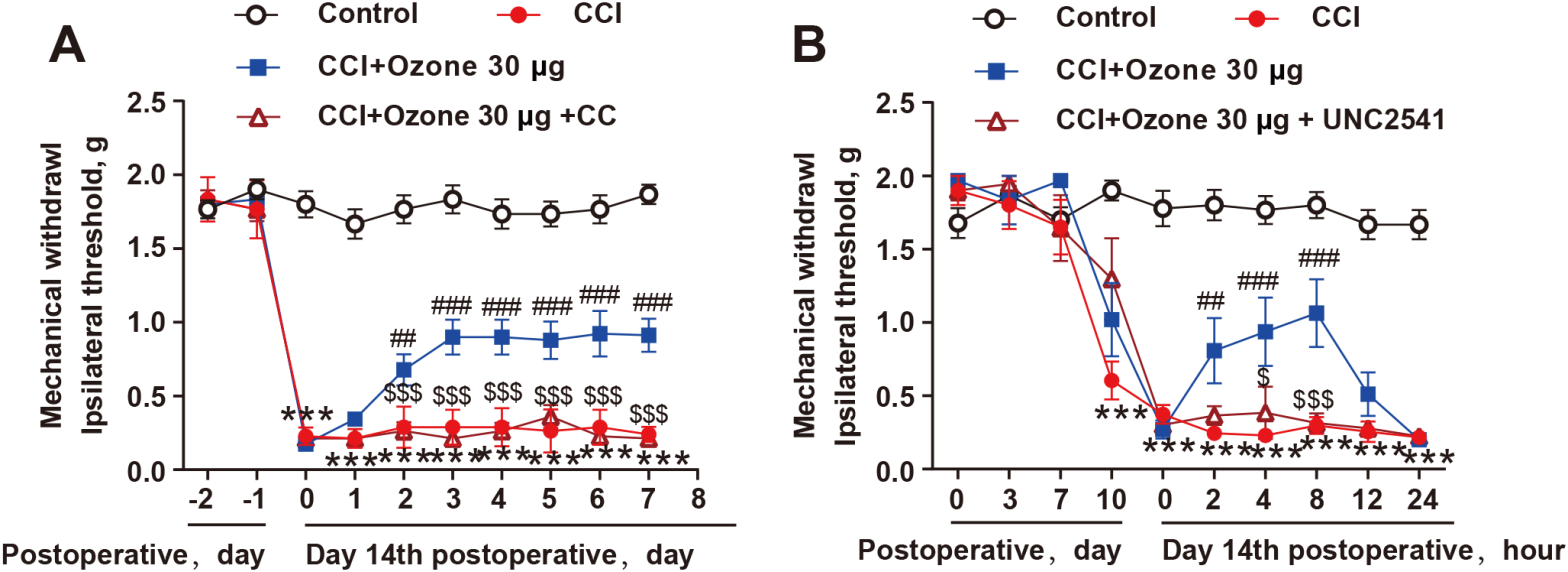
The alleviation of neuropathic pain by ozone relied on the activation of AMPK/MerTK. **(A, B)** After establishing the mouse CCI model for 14 days, mice received CC (100μg) or UNC2541 (25μM) pretreatment around the sciatic nerve half an hour in advance. Subsequently, ozone (30μg, i.p.) was administered for 7 days, and the mechanical pain threshold of mice was measured using the von Frey test (n=6). Significant differences were determined by two-way ANOVA **(A, B)**. (*p < 0.05, **p < 0.01, and ***p < 0.001 *vs*. Control; ^#^p < 0.05, ^##^p < 0.01, and ^###^p < 0.001 *vs*. CCI; ^$^p < 0.05, and ^$$$^p < 0.001 vs. CCI+Ozone 30μg.

To further validate the role of AMPK activation in promoting MerTK-mediated macrophage efferocytosis in neuropathic pain, we pre-treated mice with the AMPK inhibitor CC and the MerTK inhibitor UNC2541. The results showed that both CC and UNC2541 significantly abolished the analgesic effect of ozone in CCI mice (**Figures 6A, B**). Therefore, these findings indicated that ozone promoted macrophage efferocytosis of apoptotic cells and inhibited neuroinflammation to alleviate neuropathic pain through activating the AMPK/Gas6-MerTK/SOCS3 signaling pathway.

## Discussion

In this study, we observed pain hypersensitivity and demyelination of the sciatic nerve in CCI mice. Further investigation revealed the accumulation of apoptotic cells and inflammatory infiltration in the sciatic nerve and spinal cord, along with impaired macrophage efferocytic function. Ozone could facilitate the expression of p-AMPK/Gas6/p-MerTK, promoting the phagocytosis and clearance of apoptotic neutrophils by BMDMs. It also upregulated the expression of SOCS3 and inhibited the transcription of IL-1β, IL-6, and TNF-a, ultimately suppressing neuroinflammation and treating NPP.

Neuropathic pain is a chronic pain disorder caused by damage or diseases affecting the somatosensory nervous system. Abnormal pain and hyperalgesia are the main symptoms in patients with neuropathic pain (34). It is currently believed that changes in ion channels, activation of immune cells, neuroglia-derived mediators, and epigenetic regulation are important pathological mechanisms of neuropathic pain (2, 35). Our research indicated the accumulation of apoptotic cells and inadequate clearance leading to neuroinflammation are the main causes of neuropathic pain during the process of nerve injury (**Figure 1**). Studies have shown that TNF-α, as a key pro-inflammatory cytokine, is involved in the modulation of neuropathic pain through inducing inflammatory responses and two programmed cell death mechanisms (apoptosis and necroptosis) (36), which is consistent with our results. Ozone possesses various effects, including antimicrobial effects, immunoregulation, and antioxidant defenses. In order to verify the safety and efficacy of ozone therapy, several randomized trials were conducted in clinical settings from 1966 to 2011. Among these thousands of clinical cases, ozone therapy has shown significant effectiveness in chronic low back pain, with a low recurrence rate after cure. Ozone therapy has also been recognized in the treatment of other diseases (37). Our previous research indicated that ozone could alleviate chronic neuropathic pain induced by CCI through AMPK activation, but the specific mechanism remains unclear. Here, further research revealed that ozone inhibited cell apoptosis in CCI mice, and suppressed suppress neuroinflammatory responses (**Figure 2**). As shown in **Figure 2A**, administering ozone at different concentrations of 15μg, 30μg, and 45μg to mice, the optimal therapeutic effect was observed at a concentration of 30μg, while there was no satisfactory treatment effect at a concentration of 45μg. This point deserves our attention. Ozone is a potent oxidant and therefore has potential toxicity to organisms. Research has shown that the effects of ozone exposure are dose-dependent: high dosages stimulate severe oxidative stress resulting in an inflammatory response and tissue injury, whereas low concentrations of ozone induce a moderate oxidative eustress, activating antioxidant pathways (38). This may be the reason why ozone at a concentration of 45μg loses its therapeutic effect on CCI mice. We further measured the malondialdehyde (MDA) levels in mouse plasma to evaluate the oxidative stress levels. As shown in **Supplementary Figure S6**, the plasma MDA levels of mice treated with 15 µg and 30 µg of ozone were not significantly different from those of CCI mice. However, the plasma MDA levels of mice treated with 45 µg of ozone were significantly increased. This suggests that one of the reasons why 45 µg of ozone did not have a therapeutic effect on CCI mice is that it may have induced oxidative stress. Clinical studies have demonstrated that low-dose ozone treatment may have positive impacts in diseases such as diabetes and Parkinson’s syndrome (39). It has been reported that low doses of ozone can trigger several beneficial biochemical mechanisms and reactivate the antioxidant system. Ozone therapy can induce adaptive antioxidant and anti-inflammatory responses, showing therapeutic potential in chemotherapy-induced peripheral neuropathy (CIPN). This is similar to the clinical translational value we have found for ozone in treating neuropathic pain (17, 40). Therefore, ozone has significant clinical application prospects. However, dosage control remains an important issue during clinical use. Appropriate dosages can generate good treatment outcomes and alleviate patients’ suffering.

We further explored the mechanism by which ozone decreases the accumulation of apoptotic cells. Firstly, *in vitro*, we found ozone could promote the phagocytic clearance function of BMDMs towards apoptosis neutrophils and upregulate MerTK expression (**Figures 3A, B**). Additionally, a MerTK inhibitor, UNC2541, abolished the ozone-induced phagocytic clearance of apoptotic cells by BMDMs (**Figure 3C**). Therefore, MerTK mediated-efferocytosis played a crucial role in NPP. The mechanism by which ozone activated MerTK was further investigated. The results showed ozone could activate AMPK, upregulate Gas6 (but not Protein S). Furthermore, ozone inhibited ADAM17-mediated cleavage of MerTK, leading to increased MerTK expression and enhanced efferocytic function mediated by MerTK (**Figure 3**). This was also confirmed by live cell imaging, where BMDMs exhibited impaired phagocytic function after LPS stimulation. Ozone treatment promoted the efferocytosis of apoptotic cells by BMDMs, but this effect was abolished by the AMPK inhibitor CC (see **Supplementary Data Sheet 1**, **Supplementary Videos S1–S8**). Previous studies have shown that both Gas6 and Protein S can activate MerTK (41). However, we observed that ozone significantly upregulates Gas6, while its effect on Protein S is less pronounced. Rajotte et al. proposed that the activation of TAM receptors by Gas6 and Protein S is PS-dependent and further elucidated the importance of the structural region for Gas6-PS binding and the bridging molecule’s activation of TAM receptors (42). Literature reports indicate that in mice, MerTK receptor activation is necessary for macrophages to clear apoptotic cells, and Gas6 can enhance this process (43). This is consistent with our conclusion that ozone upregulated Gas6 to activate MerTK-mediated efferocytosis. These findings suggest that MerTK-mediated macrophage efferocytosis was crucial for the treatment of NPP and that the upregulation of MerTK by ozone relied on the activation of the AMPK/Gas6 signaling pathway, while simultaneously inhibiting ADAM27-mediated cleavage of MerTK.

The neuroinflammatory is an important factor in the development of neuropathic pain due to sensory sensitization. We found that clearance of apoptotic cells contributes to the inhibition of neuroinflammatory response primarily through upregulating SOCS3 (**Figure 4**). Our previous studies have yielded significant results regarding SOCS3 as an anti-inflammatory factor. Activation of AMPK/SOCS3 has been shown to improve chemotherapy-induced enteritis and radiation-induced enteritis (24, 44). In a morphine tolerance model, AMPK-autophagy pathway activation and miRNA-30a-5p inhibition led to upregulation of SOCS3, suppressing the neuroinflammatory response (45). We have demonstrated that promoting KATP channel opening activates the Gas6/Axl/SOCS3 signaling pathway, inducing inflammation tolerance and alleviating chronic pain (46). In this study, we further elucidated that, efficient clearance of apoptotic cells during nerve injury, which promoted SOCS3 expression, inhibited neuroinflammation.

Finally, *in vivo*, we further validated the mechanism by which ozone alleviated neuropathic pain. As shown in **Figure 5**, ozone activated AMPK, upregulated Gas6 expression (rather than protein S) in the sciatic nerve and plasma, while inhibiting ADAM17, ultimately promoting MerTK expression and facilitating macrophage engulfment of apoptotic cells, which further upregulated SOCS3 and suppressed neuroinflammation. Similarly, the AMPK inhibitor CC and the MerTK inhibitor UNC2541 were used to verify the role of AMPK and MerTK activation in ozone therapy for neuropathic pain. We found that both CC and UNC2541 abolished the analgesic effect of ozone in CCI mice (**Figure 6**).

## Conclusion

**Figure 7:**
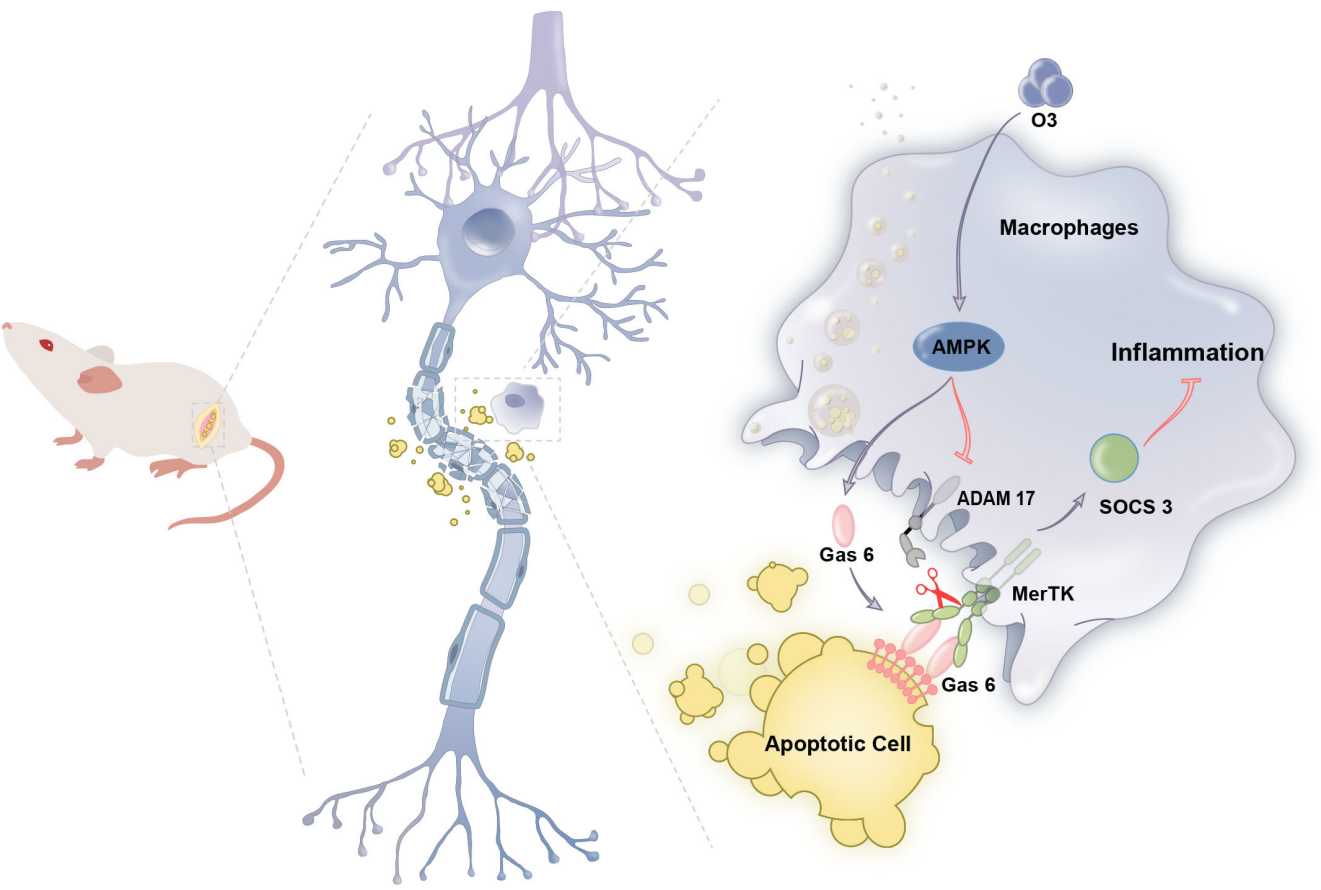
Schematic model indicates that ozone promotes macrophage efferocytosis and alleviates neuropathic pain. Ozone activities AMPK/GAS6/MerTK signaling pathway to promote macrophage efferocytosis and clear the apoptotic cells, inducing SOCS3- mediated neuroinflammation tolerance to attenuate neuropathic pain.

In summary, this research proposes for the first time that ozone enhances the phagocytosis of apoptotic cells by macrophages via the activation of the AMPK/Gas6/MerTK signaling pathway in the context of neuropathic pain. This process induces SOCS3 expression, alleviates neuroinflammation and contributes to the treatment of neuropathic pain (**Figure 7**). This study may provide effective treatment strategies for neuropathic pain while also providing theoretical support for the clinical translation of ozone therapy.

## Data Availability

The original contributions presented in the study are included in the article/**Supplementary Material**. Further inquiries can be directed to the corresponding authors.

## Supplementary material

Supplementary Figure 1 The level of Caspase3+ cells in sciatic nerve in the images of **Figure 1**. Significant differences were revealed following unpaired Student’s t-test. (*p < 0.05, **p < 0.01, and ***p < 0.001).

Supplementary Figure 2 The level of Caspase3+ cells in sciatic nerve in the images of **Figure 2**. Significant differences were revealed following One-way ANOVA. (*p < 0.05, **p < 0.01, and ***p < 0.001).

Supplementary Figure 3 Phagocytic activity of RAW264.7 Cells toward Apoptotic Neutrophils. Phagocytic activity of RAW264.7 Cells toward Apoptotic Neutrophils. The neutrophils were exposed to ultraviolet (UV) irradiation for 15 minutes to induce apoptosis. Apoptotic neutrophils were labeled with BCECF (1mg/ml) for 30 minutes. Adding the treated apoptotic neutrophils to RAW264.7 cells, co-cultivation was carried out for 15 minutes. The samples were observed under an LSM800 confocal microscope for image acquisition and analysis (n=3). Scale bars, 50μm.

Supplementary Figure 4 Hot plate test. Hot plate test. The heat pain threshold in mice was assessed using the hot plate test. The hot plate surface temperature was maintained at 53°C. The latency period, defined as the time from placing the mouse on the plate until the observation of rapid withdrawal or paw licking, was recorded. A cut-off time of 20 seconds was set. The hot test was repeated three times at 10-minute intervals, and the average value was calculated (n=6). Significant difference was revealed following two-way ANOVA. (*p < 0.05, **p < 0.01, and ***p < 0.001).

Supplementary Figure 5 The impact of the ozone-only treatment in mice. The impact of the ozone-only treatment in mice. After allowing the mice to acclimate to their environment for two days, a dose of 30 μg of ozone was administered via intraperitoneal injection once daily for nine consecutive days to investigate its effects on the mechanical pain threshold in the mice (n=6). Significant difference was revealed following two-way ANOVA. (*p < 0.05, **p < 0.01 and ***p < 0.001 *vs*. Control).

Supplementary Figure 6 MDA in plasma was detected. MDA in plasma was detected. On the 14th day after establishing the CCI mouse model, mice were exposed to ozone at different concentrations for 7 days, After establishing the mouse CCI model for 14 days, ozone (15μg, 30μg, 45μg, i.p.) was administered for 7 days. The plasma of mice was collected to measure the MDA level. Significant differences were determined by one-way ANOVA. (*p < 0.05, **p < 0.01, and ***p < 0.001).
