# Supplementary material for: Ozone promotes macrophage efferocytosis and alleviates neuropathic pain by activating the AMPK/Gas6-MerTK/SOCS3 signaling pathway
Source: Front Immunol. 2024 Nov 19;15:1455771. doi: 10.3389/fimmu.2024.1455771 (PMC11611719; doi:10.3389/fimmu.2024.1455771)
Supplement: Supplementary file 1 [file DataSheet1.docx]

Supplementary Material

**Table S1. Sequences of primers for real-time quantitative polymerase chain reaction**

| Gene |  | Primer sequences |
| --- | --- | --- |
| IL-1β | Forward | 5′-TCATTGTGGCTGTGGAGAAG-3’ |
|  | Reverse | 5′-AGGCCACAGGTATTTTGTCG-3’ |
| TNF-α | Forward | 5′-CATCTTCTCAAAATTCGAGTGACAA-3’ |
|  | Reverse | 5′-TGGGAGTAGACAAGGTACAACCC-3’ |
| IL-6 | Forward | 5′-CTGCAAGAGACTTCCATCCAG-3’ |
|  | Reverse | 5′-AGTGGTATAGACAGGTCTGTTGG-3’ |
| ACTIN | Forward | 5′-GTGACGTTGACATCCGTAAAGA-3′ |
|  | Reverse | 5′-GCCGGACTCATCGTACTCC-3′ |

**Additional Movies Figure Legends**

**Movie S1-S8.** Ozone promotes macrophage efferocytosis of apoptotic cells by activating AMPK. BMDMs were stimulated with LPS (1μg/ml) for 6 hours to establish an inflammatory model and pretreated with AICAR (300μM) or CC (20μM) for 15 minutes, followed by treatment with ozone (30μg/ml) for 4 hours. Adding the apoptotic neutrophils labeled with BCECF, the phagocytosis of apoptotic cells by BMDM was observed by Live cell workstation.

**Supplementary data Figure S1.**


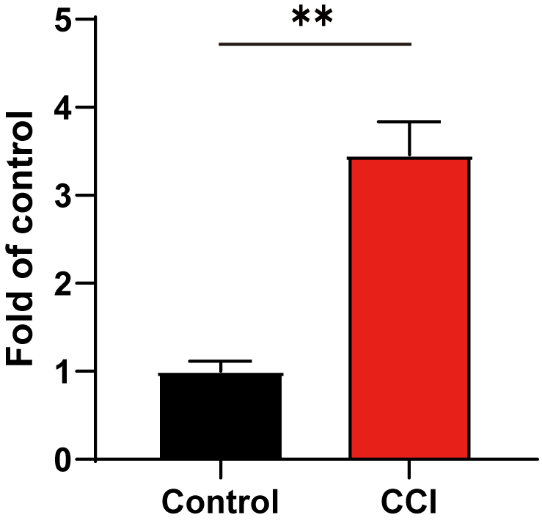


The level of Caspase3+ cells in sciatic nerve in the images of Figure 1D. Significant differences were revealed following unpaired Student’s t-test. (*p < 0.05, **p < 0.01, and ***p < 0.001).

**Supplementary data Figure S2.**

**
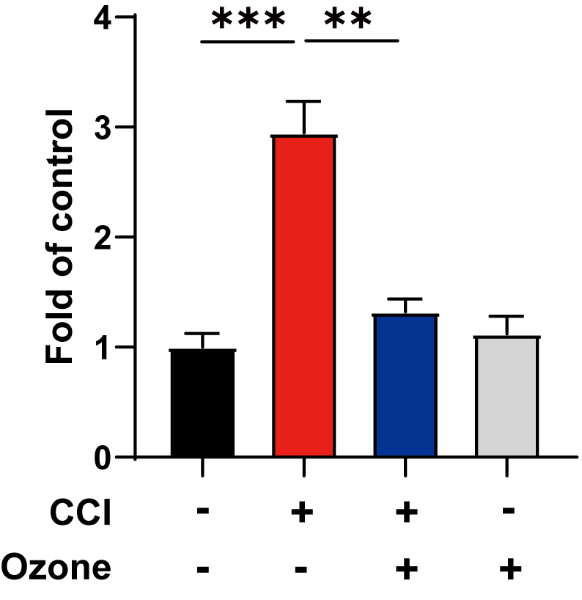
**

The level of Caspase3+ cells in sciatic nerve in the images of Figure 2D. Significant differences were revealed following One-way ANOVA. (*p < 0.05, **p < 0.01, and ***p < 0.001).

**Supplementary data Figure S3.** Phagocytic activity of RAW264.7 Cells toward Apoptotic Neutrophils.


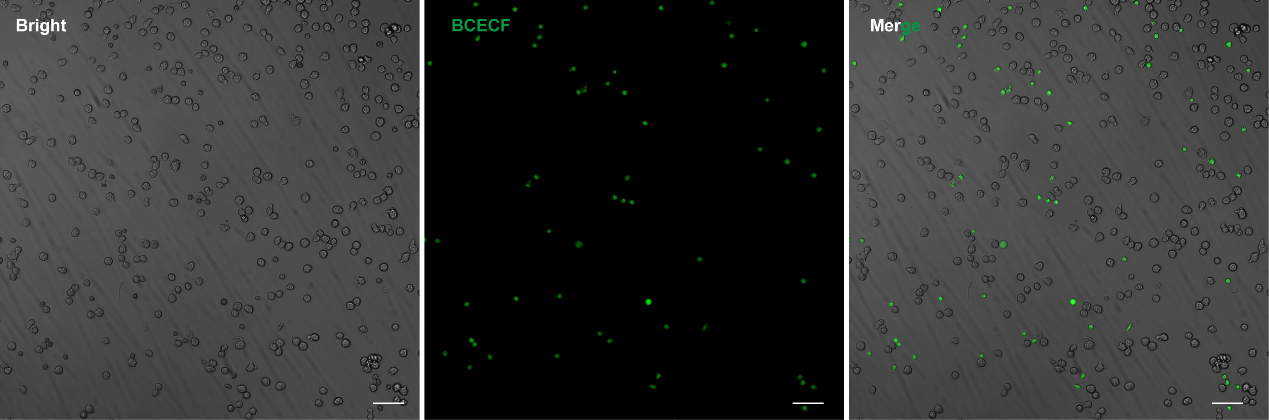


**Phagocytic activity of RAW264.7 Cells toward Apoptotic Neutrophils.** The neutrophils were exposed to ultraviolet (UV) irradiation for 15 minutes to induce apoptosis. Apoptotic neutrophils were labeled with BCECF (1mg/ml) for 30 minutes. Adding the treated apoptotic neutrophils to RAW264.7 cells, co-cultivation was carried out for 15 minutes. The samples were observed under an LSM800 confocal microscope for image acquisition and analysis (n=3). Scale bars, 50μm.

**Supplementary data Figure S4. Hot plate test.**


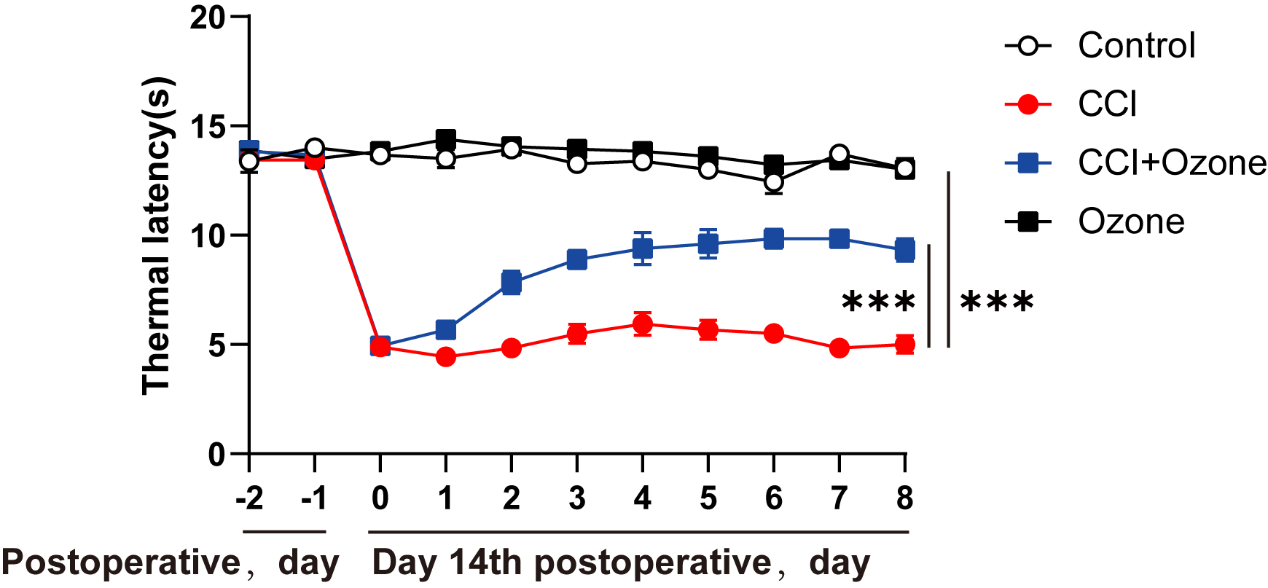


**Hot plate test.** The heat pain threshold in mice was assessed using the hot plate test. The hot plate surface temperature was maintained at 53°C. The latency period, defined as the time from placing the mouse on the plate until the observation of rapid withdrawal or paw licking, was recorded. A cut-off time of 20 seconds was set. The hot test was repeated three times at 10-minute intervals, and the average value was calculated (n=6). Significant difference was revealed following two-way ANOVA. (*p < 0.05, **p < 0.01, and ***p < 0.001).

**Supplementary data Figure S5.** The impact of the ozone-only treatment in mice.

**
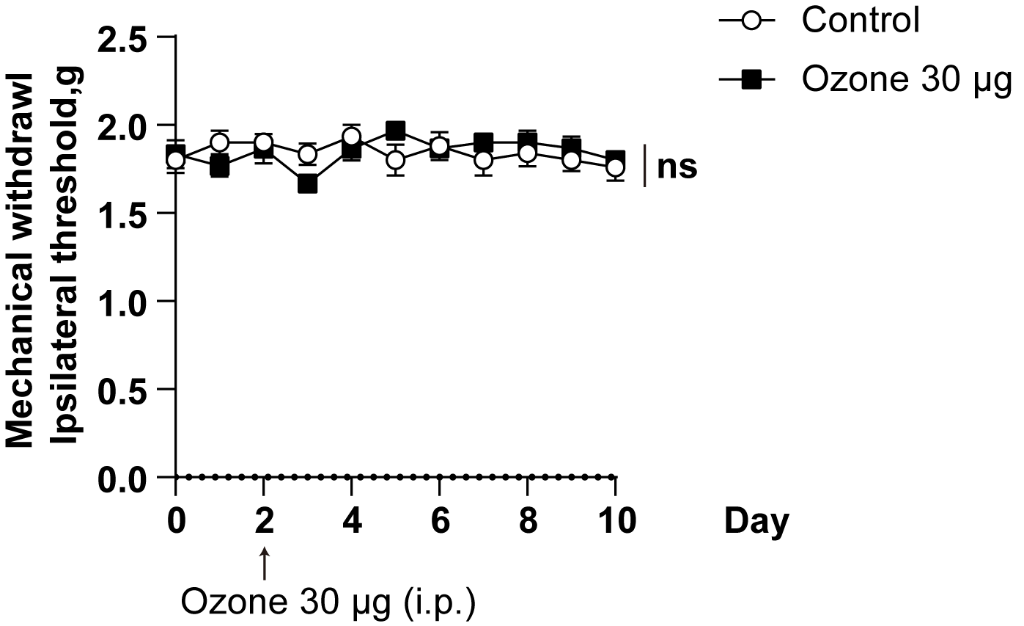
**

The impact of the ozone-only treatment in mice. After allowing the mice to acclimate to their environment for two days, a dose of 30 μg of ozone was administered via intraperitoneal injection once daily for nine consecutive days to investigate its effects on the mechanical pain threshold in the mice (n=6). Significant difference was revealed following two-way ANOVA. (*p < 0.05, **p < 0.01 and ***p < 0.001 vs. Control).

**Supplementary data Figure S6.** **MDA in plasma was detected**


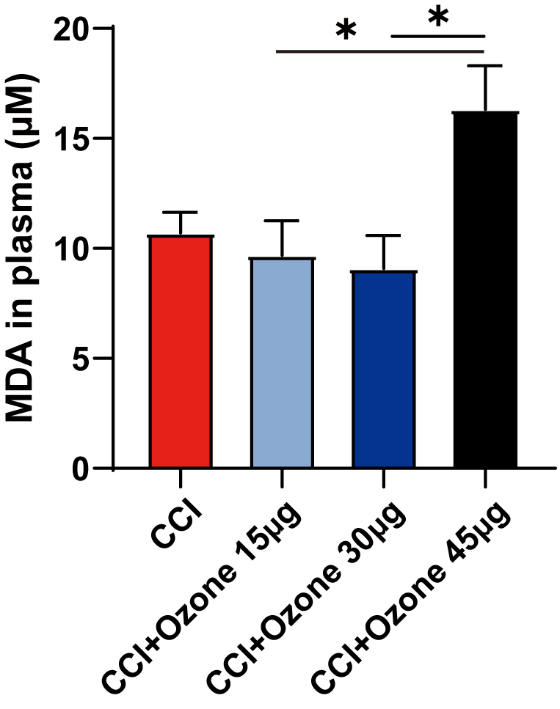


**MDA in plasma was detected.** On the 14th day after establishing the CCI mouse model, mice were exposed to ozone at different concentrations for 7 days, After establishing the mouse CCI model for 14 days, ozone (15μg, 30μg, 45μg, i.p.) was administered for 7 days. The plasma of mice was collected to measure the MDA level. Significant differences were determined by one-way ANOVA. (*p < 0.05, **p < 0.01, and ***p < 0.001).
